# Patterns of Sexual Harassment: An Intersectional Approach to Reported Victimization in a Campus Climate Survey of Students at Irish Higher Education Institutions

**DOI:** 10.1177/10778012231203619

**Published:** 2023-10-03

**Authors:** Kate Dawson, Lorraine Burke, William F. Flack, Jr, Siobhán O’Higgins, Charlotte McIvor, Pádraig MacNeela

**Affiliations:** 1 4918 University of Greenwich, London, UK; 2 8799 University of Galway, Galway, Ireland; 3 Bucknell University in Lewisburg, Lewisburg, PA, USA

**Keywords:** sexual harassment, intersectionality, college students, campus climate, minority groups

## Abstract

This study explores how identifying with multiple minority groups relates to sexual harassment victimization (SHV) among students in higher education institutions in Ireland (*n* = 6,002). Results show that gender nonconforming and female students were more likely than males to experience SHV. Bisexual or queer and gay or lesbian students were more likely than their heterosexual peers to experience SHV. Students with a physical or cognitive disability were more likely to experience SHV than those who reported no disability, and white students were more likely than minority ethnic groups to experience SHV. When controlling for sexual orientation, gender, and disability status, students who identified as both gay and lesbian and reported a cognitive disability were 8.5 times more likely to experience SHV. Victims of SHV reported having lower scores on perceived institutional support items than those who had not experienced SHV.

## Introduction

Most Irish students experience sexual harassment victimization (SHV) during their university career (MacNeela et al., 2022). In a study of 7,319 Irish university students, 67% reported experiencing some form of sexual harassment on at least 1 occasion, and 4 in 10 students reported that sexual harassment was a problem at their university (MacNeela et al., 2022). Young women make up 55% of the Irish university student body (Higher Education Authority, 2019). In Ireland, female and nonbinary students experience SHV significantly more frequently than their male peers, and LGBTQ+ students experience SHV more often than their heterosexual peers (MacNeela et al., 2022). However, no studies, in the Irish context, have explored how identifying with multiple minority group relates to SHV. The primary aim of this study is to explore how multiple minority group memberships increase the odds of experiencing SHV. Studies also show that a minority of victims report harassment events to university authorities. Consequently, there is increased attention on how third-level institutions can support victims of sexual harassment and prevent sexual harassment (SH) on college campuses. The secondary aim of this study is to explore at-risk students’ perceptions regarding their colleges’ capacity to support victims of SH. It is our intention that the findings of this study will contribute to the ongoing higher education (HE) institutional efforts to eliminate sexual harassment in Irish universities.

### Literature Review

Definitions of SH vary across sectors (Klein & Martin, 2021). However, when researching the college experience, scholars generally define SHV in terms of gender-based harassment, including offensive, hostile, and degrading comments, the use of sexist and sexual remarks and gestures, as well as sexuality-related bullying, threats, and intimidation (Fitzgerald et al., 1995). SHV also relates to unwanted sexual attention and involves behaviors that are “offensive, unwanted, and unreciprocated” (Fitzgerald et al., 1995, p. 431). Much of the research to date highlights that women and sexual minority groups are more often the victims of sexual harassment, with many reporting being victimized by men (Fitzgerald & Cortina, 2018).

A significant amount of student SHV happens on college campuses. Several studies have shown that students report being sexually harassed in student common areas, campus accommodation, lecturers’ offices, lecture halls, or classrooms (Clodfelter et al., 2010; Hill & Silva, 2005; Owoaje & Olusola-Taiwo, 2010). Harassment is associated with negative mental and physical health outcomes, including increased psychological distress, posttraumatic stress disorder (PTSD), depression, increased social isolation, and feelings of helplessness, internalized shame, problematic alcohol use, and sleep disturbances (Dawson et al., 2023; Huerta et al., 2006; Shinsako et al., 2001; Street et al., 2007). Students who experience SHV during their time in college also report reduced academic achievement, including college avoidance, feelings of insecurity on campus, and elevated risk of program noncompletion (Huerta et al., 2006; Mellgren et al., 2018; Rosenthal et al., 2016).

There is a large body of evidence that shows women and other minority groups, regardless of the culture or context, are disproportionately affected by SHV (Arulogun et al., 2013; Bondestam & Lundqvist, 2020; MacNeela et al., 2022; Owoaje & Olusola-Taiwo, 2010; Rosenthal et al., 2016; Yoon et al., 2010). For instance, studies on public transit crimes indicate that minority groups are at increased risk of SHV than other groups (Ludici et al., 2017, 2019; Santoro et al., 2020). Women and minority groups are also more likely to be sexually harassed in the workplace (Cortina & Areguin, 2021). Until recently, characteristics associated with SHV have largely been explored in terms of individual predictors. Recently, studies have begun to explore SHV in terms of cumulative risk.

A recent review of the literature identified several significant gaps in SHV research with students (Bondestam & Lundqvist, 2020). Most notably, there is limited research regarding SHV of students outside of the United States. Much of the research describes the experiences of white, heterosexual, and cisgender women—a relatively privileged group in comparison with other women and gender and sexual minorities. The review also highlighted a dearth of studies SHV among minority groups and studies that employ an intersectional approach to better understand SHV from the perspective of equality and inclusion (Bondestam & Lundqvist, 2020).

The evidence suggests that particular groups experience higher rates of SHV, therefore inviting further exploration of these patterns and intersections between risk factors. There is a broad gender effect for example, with women reporting higher rates in surveys. In addition, membership of minority groups such as ethnic, gender, and sexual minorities and disability is associated with elevated risk of SH. Incidence rates of SHV are higher among students with disabilities than those without disabilities (Cantor et al., 2019; Plummer & Findley, 2012) and are higher among LGB+ students compared with their heterosexual peers (Boyle & McKinzie, 2018). Research often focuses on the SHV outcomes associated with individual characteristics such as gender or sexual orientation, yet, in reality, individuals inhabit multiple identities at once.

In the university context, a Canadian study showed that women with a disability were at greater risk of SHV than other women (Stermac et al., 2018). In addition, Boyle and McKinzie (2018) found higher rates of SHV among female LGB+ students in comparison with heterosexual females. Similar findings are observed in Brazil. Nourani et al. (2020) concluded that LGBT+ women were the most victimized group of in-transit university students. Such findings suggest that university students who inhabit intersecting identities are at risk of SHV in the settings in which they attend.

#### Institutional support

The second area of enquiry for this study is to examine at-risk groups’ perceptions regarding their university institutions’ capacity to support victims of SHV. Institutions have the capacity to support victims of sexual harassment but also can cause additional harm to assault survivors (Mueller et al., 2001; Smith & Freyd, 2013). The term “institutional betrayal” is used to describe how trusted institutions act in a way that creates or reinforces harm on those dependent on them for their safety and wellbeing (Smith & Freyd, 2014). In a university context, Smith and Freyd (2013) found that victims of sexual harassment and other sexual crimes commonly report institutional betrayal and were associated with increased levels of anxiety, trauma-specific sexual symptoms, dissociation, and problematic sexual functioning (Smith & Freyd, 2013). Their findings are supported by multiple studies conducted across different contexts (Fox & Tang, 2017; National Academies of Sciences, Engineering, and Medicine, 2018; Raver & Nishii, 2010).

The current study findings will help to identify specific areas of focus for intervention within Irish college communities.

### Theoretical Framework

The current study examines SHV within an intersectional framework, by examining the individual and contextual factors that place an individual at increased odds of SHV within the college environment. Taking a feminist and critical race theory perspective, Crenshaw (1989) and McCall (2005) argued that a single-axis framework for exploring specific outcomes sheds light on those who are disadvantaged in relation to those who are privileged, for example, LGB+-identifying versus heterosexual and female versus male. While bringing access to power and resources into perspective, the single-axis framework is incomplete given that forms of oppression linked to individual characteristics act in concert rather than independently (Crenshaw, 1989; Essed, 1991). Therefore, a single variable like race, gender, or sexuality may lack nuance in documenting and understanding SHV experiences, including providing insights into perpetrator targeting of individuals for harassment. Considering that race, gender, disability, and sexuality are each risk factors for SHV, the adverse effects of racism, sexism, homophobia, and ableism might combine in some contexts. The concept of intersectionality considers the combination of these factors and has been defined as the “multidimensionality of marginalised groups” (Crenshaw, 1989, p. 139).

As the limited evidence available on the topic suggests, combining categories such as LGB+, women, or gender nonconforming identity with a disability is likely to be reflected in experiences of SHV (Jackson & Williams, 2006). It is for these reasons that there has been a call, in recent years, to explore victimization in terms of intersecting identities, rather than positioning risk in terms of independent groups. However, often limited due to study sample size, few studies have quantitatively explored SHV in terms of intersecting groups and, to the best of the authors knowledge, no study has quantitatively explored these associations outside of Canadian and the American universities (Boyle & McKinzie, 2018; Stermac et al., 2018; Wood et al., 2018).

Universities are an appropriate environment to explore SHV for several reasons. A growing body of literature suggests that universities, and indeed institutions more generally, can increase or decrease the likelihood of sexual offending (Gantman & Paluck, 2022).

Gantman and Paluck's theoretical framework describe how individual factors, e.g., internal mental processes, and situational factors interact to predict sexual violence. The college environment presents an appropriate case study in which to explore the interplay between these factors as studies consistently observe trends in increased frequency in sexual behavior, isolation from one's home community, and frequent socializing involving drug and alcohol use, in the college environment (McGraw et al., 2022).

### Cultural Context

This study explores experiences of SHV among students in the Irish HE sector, which helps address the gap in research outside the United States (e.g., Swartout et al., 2019) and recent calls for enhanced surveillance of SVH in European HE. The European Research Area and Innovation Committee (ERAC) Standing Working Group on Gender in Research and Innovation (2019) has called on European Union (EU) member states to be increasingly proactive in ensuring equal access to education and to an equal learning environment while enrolled in HE. This includes the provision of specific needs–based support to disadvantaged groups and the removal of structural and system barriers to education (ERAC Standing Working Group on Gender in Research and Innovation‌, 2019). SHV is an important threat in this context, considering the risk of SHV among disadvantaged and minority groups, the significant impact of SHV on the wellbeing of students, and its role negatively impacting educational achievement. It is therefore important to identify the risks for student groups, the confluence of risk factors highlighted through an intersectional approach, as well as the necessary institutional policies, actions, and research needed to support those who experience SHV.

The current study is the first of its kind in Ireland. It uses a quantitative survey design, analyzing the findings of a recent national campus climate survey to explore SHV experiences among students living in Ireland. The focus of the enquiry is in terms of categories highlighted in previous research, namely gender, sexuality, disability, and ethnic backgrounds. We then explore how multiple minority group memberships relate to the risk for SHV.

Research questions

1. Are gender, sexuality, ethnicity, and disability risk factors for SHV in Irish colleges?
2. Does the intersection of specific risk factors further increase the likelihood of SHV in Irish colleges?
3. Do at-risk groups for SHV have lower scores regarding perceived institutional support?

## Methodology

### Design

The current study provides a cross-sectional overview of SHV experiences of students in Irish colleges through an anonymous online survey.

### Participants

In total, 6,002 of the 225,600 registered students within Irish HE completed the survey (Higher Education Authority, 2019). Participants were recruited from a broad range of disciplines and at various levels of third level education. Of the 21 HEIs invited to participate, 14 sent out the invite to students on their campus, consisting of 10 institutes of technology and 4 universities. The majority was aged 18–24 (83%), identified as heterosexual (74%), white Irish (80%), with no disability (85.5%), and enrolled in an undergraduate degree program (89%). See Table 1 for participant demographic characteristics.

**Table 1.** Participants’ Demographic Information by Gender *n* (%).

	Nonbinary	Female	Male	Total
Age				
18–24	68 (78.2)	3,388 (86.3)	1,502 (75.6)	4,958 (82.6)
25+	19 (21.8)	540 (13.7)	485 (24.4)	1,044 (17.4)
Sexual orientation				
Asexual	5 (6)	179 (5)	84 (4)	268 (4.5)
Bisexual	26 (29.5)	635 (16)	159 (8)	820 (14)
Gay	9 (10)	10 (.3)	160 (8)	179 (3)
Heterosexual	7 (8)	2,904 (74)	1,540 (77.5)	4,451 (74)
Lesbian	6 (7)	65 (2)	1 (0)	72 (1)
Queer	18 (20.5)	44 (1)	4 (.2)	66 (1)
Other	16 (18)	60 (1.5)	25 (1)	101 (2)
Disability status				
No disability	34 (39.1)	3,293 (83.8)	1,803 (90.7)	5,130 (85.5)
Specific learning difficulty	2 (2.4)	59 (1.5)	27 (1.4)	88 (1.5)
Physical or mobility-related	4 (4.8)	25 (0.6)	9 (0.5)	38 (0.6)
Visual impairment	0	6 (0.2)	4 (0.2)	10 (0.2)
Hearing impairment	0	10 (0.3)	5 (0.3)	15 (0.3)
Mental health difficulty	31 (36.9)	399 (10.3)	64 (3.3)	494 (8.3)
ASD or Asperger's	8 (9.5)	18 (0.5)	29 (1.5)	55 (0.9)
ADHD or ADD	1 (1.2)	17 (0.4)	11 (0.6)	29 (0.5)
Ongoing physical illness	4 (4.8)	59 (1.5)	13 (0.7)	76 (1.3)
Race/ethnicity				
White–Irish	62 (76.5)	3,078 (80.5)	1,536 (79.6)	4,676 (80.2)
White–Irish traveler	1 (1.2)	10 (0.3)	8 (0.4)	19 (0.3)
White–any other white background	13 (16.0)	530 (13.9)	215 (11.1)	758 (13.0)
Black or black Irish–African	1 (1.2)	65 (1.7)	45 (2.3)	111 (1.9)
Black or black Irish–Any other black background	0	10 (0.3)	5 (0.3)	15 (0.3)
Asian or Asian Irish–Chinese	2 (2.5)	34 (0.9)	24 (1.2)	60 (1.0)
Asian or Asian Irish–any other Asian background	2 (2.5)	95 (2.5)	96 (5.0)	193 (3.3)
Undergraduate year				
First	31 (39.7)	1,272 (35.8)	631 (36.9)	1,934 (36.2)
Second	13 (16.7)	982 (27.6)	442 (25.8)	1,437 (26.9)
Third+	34 (43.6)	1,301 (36.6)	638 (37.3)	1,973 (36.9)
Postgraduate course				
Postgraduate taught	6 (66.7)	274 (73.5)	202 (73.2)	482 (73.3)
PhD/research masters	3 (33.3)	99 (26.5)	74 (26.8)	176 (26.7)
Total	72	3,868	1,961	6,002

*Note*: ASD, autism spectrum disorder; ADHD, attention deficit hyperactivity disorder; ADD, attention deficit disorder

### Measures

The online survey was based on the ARC3 Campus Climate Survey (Swartout et al., 2019), a comprehensive module-based survey tool that has previously been tested which incorporates a number of validated measures (Swartout et al., 2019). The survey form was adapted to the Irish HE sector (e.g., changes to vernacular) through a review process by the researchers informed by student panels and the national student advocacy body (the Union of Students in Ireland).

Demographic questions included gender (“What is your gender identity?”), sexual orientation (“What is your sexual orientation?”), disability (“Do you have a disability including a mental or physical illness?”) with response options 0 = yes and 1 = no, and disability type (“What is your disability?”). Students who indicated a disability were then provided with follow-up response options to choose from to indicate which disability(s) they had (see Table 1). Visible disabilities may be disproportionately targeted for harassment than those with nonvisible disabilities or no disability (Scherer & Reyns, 2019). Therefore, disability status responses were aggregated into three categories: no disability, physical disability, and cognitive/mental health disability. Students were provided with the following response options regarding gender identity: female, male, transgender, gender nonconforming, nonbinary, and prefer not to say. Gender identity responses were aggregated into three categories to improve statistical power: female, male, and gender nonconforming. The following response options were provided for sexual orientation: asexual, bisexual, gay, heterosexual/straight, lesbian, queer, and prefer not to say. Responses were aggregated for the purpose of analysis into LGBQ+ and heterosexual individuals.

### Dependent Variables

#### Sexual harassment

Sexual harassment was assessed using 12 items, each with a 5-point response scale (0 = never to 4 = many times). Items related to sexual harassment in college, including sexist hostility, sexual hostility, and persistent attempt to initiate a relationship (items 1–9), were sourced from the ARC3 Campus Climate Survey (Swartout et al., 2019), which were in turn modified from the Sexual Experiences Questionnaire (Fitzgerald et al., 1988, 1995). These items used to explore incidence of SHV since the beginning of college have shown high internal consistency as part of the ARC3 Campus Climate Survey (Swartout et al., 2019). Scoring items 9–12 assessed experiences of online sexual harassment and have demonstrated strong internal consistency (Nukulkij, 2011). For the purpose of analysis, a combined variable, consisting of the 12 SHV items, was created. Response options were also recoded into a binary response option (0 = no experience of SH and 1 = any experience of SH).

#### Follow-Up Questions on Sexual Harassment

##### Perpetrator Student Status

Students were asked to reflect on one SHV event that had the greatest effect on them and were asked if the perpetrator was a student at their university or college. Response options were yes, no, and don’t know.

##### SHV Location

Students were asked to reflect on one SHV event that had the greatest effect on them and were asked “Did this event happen on campus?” to which the responses were coded 0 = yes and 1 = no.

#### Perceived Institutional Support

Perceptions of institutional support measures were derived from Rutgers Campus Climate Survey, which is a further ARC3 survey module 4 (de Heer & Jones, 2017; Swartout et al., 2019). This measure comprised six items about students’ perception of how the college would respond to an incident of sexual misconduct.

### Independent Variables

#### Procedure

##### Sampling and Recruitment

College administrators were initially contacted by email, providing background information to the study and inviting each institution to participate. On agreement, college administrators then forwarded the study invitation to all registered students which contained a link to the online survey. The study had intended for full national coverage but was interrupted by COVID-19 public health restrictions. Students were also invited to take part in the study through each of the 14 institutions’ existing students’ union. The Union of Students in Ireland (USI) was a national partner to the researchers on the campus climate survey project and provided input to student review of the cultural relevance of the questions and by advertising the study on a national level directing students to the survey link on their website. The first page of the survey provided participants with further information about the details of the study. Support service information was also provided to students before they began answering the survey items. Students and college administrators were informed that individual institution study findings would not be reported back to the institution or in any official publications arising from the study. Students were informed that they must be aged 18 years or older to take part in the survey. The survey was incentivized, and students who completed the questionnaire were given the option to enter a prize draw for a voucher. Respondents were assured that the email address they submitted to enter the draw could not be connected with their survey responses.

All students who received the invitation to participate in the survey and/or partially or fully completed the questionnaire were provided with support service information. Approximately 12,000 students began the survey. In total, 6,002 completed it, with an average response time of 23 min. This study received ethical approval from the research ethics committee at National University of Ireland (NUI) Galway.

#### Analysis

##### Experience of SHV

First, we report the percentage scores for each combined group regarding their experiences of SHV (Table 2)—for example, for the purpose of analysis in Model 1, all cognitive and mental health–related disabilities reported were combined to produce the item “cognitive disability.” As SHV variables are categorical, chi-square statistics are reported along with Cramer's *V*. We then present two logistic regression models.

**Table 2.** Experience of Sexual Harassment Victimization by Demographic Characteristics *n* (%).

	No SHV	Any SHV	Total	χ^2^ Cramer's *V*
Gender**				46.95 (.09)
Female	888 (23)	3,040 (77)	3,928	
Male	592 (30)	1,394 (70)	1,986	
Nonbinary	9 (10)	78 (90)	87	
Sexual orientation**				61.04 (.11)
Bisexual/queer	129 (15)	757 (85)	886	
Gay/lesbian	54 (21)	197 (79)	251	
Heterosexual	1,193 (27)	3,258 (73)	4,451	
Disability status**				51.11 (.09)
Physical disability	14 (10)	125 (90)	139	
Cognitive disability	106 (16)	560 (84)	666	
No disability	1,370 (26)	3,827 (74)	5,197	
Ethnicity**				79.95 (.11)
White	1,277 (23.4)	4,176 (76.6)	5,453	
Black	47 (37)	79 (63)	126	
Asian	117 (46)	136 (54)	253	
Other	49 (29)	121 (71)	170	
Total	1,490 (25)	4,512 (75)		

**p < .01.

##### Follow-Up Questions on SHV Event

We asked two follow-up questions about the SHV event that had the greatest effect on the participant. The first question pertained to the student status of the other person, and the second pertained to the location in which the event took place. We present frequencies and percentages for the overall sample in Table 3.

**Table 3.** Follow-Up Questions on Experience of Sexual Harassment Victimization for Total Sample.

Question	*n* (%)
Was the other person a student at your college?	
Yes	2,116 (46.9)
No	1612 (35.7)
I don’t know	784 (17.4)
Did this happen on campus?	
Yes	1,154 (25.6)
No	3,358 (74.4)

##### Basic Model

The first presents our basic model consisting of four independent variables: sexual orientation (three levels), gender (three levels), disability status (three levels), and ethnicity (four levels). The dependent variable consisted of two levels, no experience of sexual harassment and experience of sexual harassment (Table 4).

**Table 4.** Sexual Harassment Victimization Likelihood by Sexual Orientation, Gender Identity, Disability Status, and Ethnicity.

	B	SE	Wald	Df	Sig.	Exp(B)
Male			23.060	2	<.001	
Nonbinary/nonconforming	.705	.413	2.915	1	.088	2.024
Female	.308	.066	21.536	1	<.001	1.361
Heterosexual			35.896	2	<.001	
Bisexual/queer	.602	.105	33.084	1	<.001	1.826
Gay/lesbian	.344	.162	4.503	1	.034	1.410
No disability			16.907	2	<.001	
Physical disability	.913	.297	9.462	1	.002	2.492
Cognitive disability	.339	.118	8.169	1	.004	1.403
White			56.377	3	<.001	
Black	−.654	.196	11.122	1	<.001	.520
Asian	−.950	.141	45.401	1	<.001	.387
Other minority group	−.307	.183	2.794	1	.095	.736
Constant	.845	.055	233.659	1	<.001	2.327

##### Interaction Model Development

The second model builds upon Model 1 to include the relevant interaction terms (gender × sexual orientation, gender × disability status, gender × ethnicity, sexual orientation × disability status, and sexual orientation × ethnicity). Ideally, we would explore all interactions between all variables in our basic model. However, we were limited in terms of statistical power in conducting analyses among smaller groups. For example, when we explored frequencies among those who reported being black and having a disability, one black student reported having a visible disability, and five reported having an invisible disability. Similarly, 18 black students identified as being LGB+. Recent reports identified that 2.4% of the student body in Ireland identify as being black (Higher Education Authority, 2019). It was not feasible to explore interaction terms with asexual, gender nonconforming participants, black, Asian, or other minority ethnic groups in this analysis, due to the small number of participants in each group (Table 5).

**Table 5.** Sexual Harassment Victimization Likelihood by Sexual Orientation, Gender Identity, Disability Status, Ethnicity, and Interaction Terms.

	B	SE	Wald	df	Sig.	Exp(B)
Heterosexual			9.880	2	.007	
Bisexual/queer	.659	.221	8.876	1	.003	1.933
Gay/lesbian	.236	.197	1.431	1	.232	1.266
Female	.330	.072	21.139	1	<.001	1.391
No disability			4.499	2	.105	
Physical disability	1.444	.686	4.433	1	.035	4.239
Cognitive disability	.074	.239	.096	1	.757	1.077
Heterosexual × male			.191	2	.909	
Bisexual/queer × female	−.101	.247	.168	1	.682	.904
Gay/lesbian × female	.049	.379	.016	1	.898	1.050
Male × no disability			.963	2	.618	
Female × physical disability	−.360	.741	.236	1	.627	.698
Female × cognitive disability	.227	.271	.701	1	.402	1.255
Heterosexual × no disability			8.067	4	.089	
Bisexual/queer × physical disability	−.392	.720	.297	1	.586	.676
Bisexual/queer × cognitive disability	.292	.287	1.039	1	.308	1.340
Gay/lesbian × physical disability	−1.752	1.059	2.738	1	.098	.173
Gay/lesbian × cognitive disability	2.147	1.044	4.227	1	.040	8.559
Constant	.762	.056	184.031	1	<.001	2.142

##### Final Interaction Model

The final interaction model included the revised variables from Model 1: gender, two levels (female and male); sexual orientation, three levels (bisexual/queer, lesbian/gay, and heterosexual); and disability status, three levels (physical disability, cognitive disability, and no disability) (Table 5).

Heterosexual, male students, without a disability were selected as the reference groups within their respective categories because of their previously documented lower relative risk for SHV in comparison with their peers (Bondestam & Lundqvist, 2020; Boyle & McKinzie, 2018; Cantor et al., 2019; Plummer & Findley, 2012).

##### Institutional Support

To assess students’ perception of institutional support for victims of SH, six items were combined to produce one continuous variable (a = .85). We conducted a series of T-tests and ANOVAs to examine the mean differences among those who experienced SHV and those who did not, the groups at higher risks of SHV, and those who experienced their most severe form of SHV on campus, on their perceived institutional support scores.

## Results

### Model 1: Basic Model

A logistic regression was performed to ascertain the predictive effect of race, gender, sexual orientation, and disability status on SHV. The model was statistically significant [χ^2^(9) = 175.38, *p* < .001] and explained 5% of the variance.

#### Gender

Women were 1.4 times more likely than men, and gender nonconforming individuals were 2 times more likely than men to be victims of sexual harassment.

#### Sexual Orientation

Bisexual or “queer” identifying individuals were 1.8 times more likely to be victimized than heterosexual-identifying individuals. Gay or lesbian identifying individuals were 1.4 times more likely to be victimized than their heterosexual peers.

#### Disability Status

Having a physical disability increased the risk of SHV by 2.5 times in comparison with those who did not have a disability. Having a cognitive/mental health disability increased the risk of SHV by 1.4 times in comparison with those without a disability.

#### Ethnicity

Being white was a significant risk factor for SHV. Being black reduced the risk of SHV by 0.5 times in comparison with being white. Being Asian reduced the risk of SHV by 0.4 times in comparison with being white. Being another minority group apart from black or Asian reduced the risk of SHV victimization by .7 in comparison with being white (see Table 3).

### Model 2: Interaction Terms

Interaction terms were created to explore the interaction of gender, sexual orientation, and disability status on the likelihood of SH victimization. The model was statistically significant [χ^2^(13) = 123.80, *p* < .05] and explained 3% of the variance. When controlling for sexual orientation, gender, and disability status, the interaction effect between identifying as gay or lesbian and reporting a cognitive disability was statistically significant and were 8.5 times more likely to experience SHV than their peers. No significant interactions were found between gender and sexual orientation or disability status or between sexual orientation and the other disability groups.

### Perceived Institutional Support

When assessing perceived institutional support, in addition to reporting the overall percentages within the total sample, we also chose to assess perceived institutional support among the groups identified as being most at risk for SHV in Model 1 and Model 2.

#### Institutional Support Overall

Perceived institutional support was high overall with a majority reporting that it was likely or very likely that the college would take the SHV report seriously (78%), maintain the privacy of the victim (86%), take steps to protect the victim (77%), take action to address factors that led to sexual misconduct (64%), and handle the report fairly (72%). A minority believed that the college would label the victim a troublemaker (9%) and punish the person making the report (6%).

#### Institutional Support by Group

A T-test and series of ANOVA were conducted to explore the mean differences between students who experienced SHV and those who did not and the various groups included in Model 1 regarding their perception of their institutions’ cultural practice of supporting victims of SH.

#### Sexual Harassment Victimization

There was a significant difference in perceived institutional support agreement levels between students who experienced SHV (M = 15.71, SD = 2.77) and those who had not [M = 16.79, SD = 2.11, *t*(3314.45) = 15.76, *p* < .001], *d* = .44. Those who experienced SHV reported less perceived institutional support from their institution than those who had not experienced SHV.

#### Gender

There was a significant difference between nonbinary and gender nonconforming (M = 14.93, SD = 3.18), female (M = 15.90, SD = 2.73), and male students (M = 16.17, SD = 2.49) regarding perceived institutional support [*F*(2, 5999) = 13.60, *p* < .01]. Men perceived the greatest institutional support, and nonbinary students reported the lowest scores regarding perceived institutional support.

#### Ethnicity

There was no significant difference between different ethnic groups regarding their perceived institutional support [*F*(3, 5998) = 2.17, *p* = .09].

#### Disability Status

There was a significant difference between students with physical (M = 15.32, SD = 3.05), cognitive, or mental health disabilities (M = 15.22, SD = 3.22) and students who reported no disability (M = 16.09, SD = 2.56) regarding perceived institutional support [*F*(2, 5999) = 35.85, *p* < .001]. Students with no reported disability reported the highest scores regarding perceived institutional support.

### Location of SHV and Institutional Support

There was no significant difference in the scores between those who experienced their most severe form of SHV on campus (M = 19.55, SD = 3.88) and off campus (M = 19.38, SD = 3.70), on their reported perceptions of institutional support *t*(4510) = 1.326, *p* = .185.

## Discussion

This study provides exploratory data into identity-based factors associated with SHV among students living in Ireland. Several studies have highlighted that women are more likely to experience SHV in comparison with men (Bondestam & Lundqvist, 2020; Fitzgerald & Cortina, 2018; MacNeela et al., 2022). Fewer studies have highlighted the SHV risk associated with identifying with multiple minority groups (Cortina & Areguin, 2021; Ludici et al., 2017; Santoro et al., 2020). Our findings support others who have shown that, as well as gender, sexual orientation, ethnicity, and disability status are important predictors for SHV (Boyle & McKinzie, 2018; Stermac et al., 2018). In addition, the study showed that groups most at risk of SHV tended to report lower scores regarding perceived institutional support.

Building on work by Nourani et al. (2020), Stermac et al. (2018), Boyle and McKinzie (2018), Wood et al. (2018), and Ludici et al. (2017), our findings suggest that multiple minority category memberships representing intersectional identity increases the odds of experiencing SHV. Specifically, our study provides additional novel findings indicating that, in Irish HE institutions, identifying as both gay or lesbian and having a cognitive disability places someone at particular risk of SHV. To the best of the authors’ knowledge, this is the first study to quantitatively demonstrate this finding. This indicates that, in this particular context, individuals in more than one minority group risk being victims of multiple levels of social injustice (Boyle & McKinzie, 2018; Stermac et al., 2018; Wood et al., 2018). Our findings support previous studies that show using an intersectionality framework is appropriate when examining SH experiences, as individuals with multiple identifying characteristics experience sexual harassment concurrently (Boyle & McKinzie, 2018). However, the cross-sectional nature of our study limits our ability to determine the direction of our findings. It may be that gay or lesbian individuals experience cognitive or mental health–related difficulties due to their experiences of harassment, which indeed is well documented to be associated with psychological ill health (Huerta et al., 2006; Shinsako et al., 2001; Street et al., 2007).

The findings are particularly concerning considering the lower perceived institutional support among these at-risk groups. The findings add to the growing body of literature that highlights the consequences associated with being outside of the circle of privilege (Fox & Tang, 2017; Neupane & Chesney-Lind, 2014; Smith & Freyd, 2013). However, it is also important to note that Model 1 explained only 5% of the variance of SHV and, within our sample, the findings show little evidence of intersectionality as the main feature of patterns of SHV, over and above minority group membership. This does not mean, however, that our nonsignificant findings related to SH risk among certain combinations of identities default to similarity with other groups. For example, experiences of LGB+-identifying women and women who report having a disability may differ. There are multiple categories, psychological experiences, and behaviors that differ between individuals who experience SHV. Further qualitative research is needed to explore how SHV experiences for those with intersecting minority group memberships may differ to others in terms of factors such as reporting, peer support, or psychological impact. However, the small percentage of variance explained by our regression model may indicate that although characteristics may partially explain why an individual may be targeted for SH, it is likely that SH is explained by additional factors including the motivations of the perpetrator (Cortina & Areguin, 2021).

Being white was associated with increased risk for SHV. This contradicts findings from other countries that have identified people of color at increased risk of SHV—see Cortina and Areguin (2021) for review. However, Ireland is a predominantly white country. The issue of common-bond association between the harasser and victim may help to explain these findings. Common bond is used to justify SH. Richardson and Taylor (2009) posit that perpetrators of SH are more likely to believe that an individual with shared characteristics, for example, race, would be less likely to take offense to harassing behaviors (Richardson & Taylor, 2009), thus minimizing the perceived severity of their harassing behavior. Exposure to SHV may also be linked to lifestyle factors, including living situation, social groups, alcohol, and drug use (McGinley et al., 2016). For example, McGinley et al. (2016) found that society or club-related involvement at college was associated with both generalized and sexual harassment and was particularly high for female students.

In addition, a victim's lived experience of social categories may influence their perception of the motive for the harassing behavior. For example, Buchanan and Ormerod (2002) found minority women described SHV experience in terms of racial and sexist harassment, while white women only referenced sexism, perhaps because white women are less likely to consider racism as an impetus for harassment. Stereotypes about certain groups also influence sexual harassment victims’ perceptions regarding the reason they were harassed and may indeed explain why some individuals are targeted for harassment. For example, in one study, Hispanic women reported “[Harassers] will say that they hear that Hispanic women are good in bed or whatever and they just go after it” (Richardson & Taylor, 2009). It was beyond the remit of this study to explore perpetrators’ justification for their SH behavior; however, the prevalence of SH among students may be partially explained by the fact that perpetrators of SH often do not perceive their behavior as harassing. For example, Dougherty (2001) found that some men believed that engaging in sexually harassing behavior was a way of expressing camaraderie in the workplace, while women reported that sexual harassment isolated them from their coworkers and made them feel uncomfortable.

### Institutional Support

Decision-making regarding experiences and reporting of harassment are influenced by the social construction of race, gender, orientation, and disability status in college (Decker et al., 2019). We found that some minority groups were less likely to believe that their institution would deal with an allegation of SH effectively. This may be because of having a direct experience of dealing with college support services or through experiencing inadequate support elsewhere. We found that experiencing ones’ most severe form of SHV on campus was not significantly associated with students’ perceptions regarding their institutions capacity to support victims of SHV. However, the finding relates to *one* experience of SHV only, when individuals may have experienced SHV on several occasions. In addition, we do not know if student views about their institution contribute to or act as a protective factor against SHV-related university experience. Previous studies using the same measures have identified perceived quality of institutional support was positively associated with more positive beliefs around the impact of reporting SHV (Swartout et al., 2019).

Perceptions of an institution's likelihood to respond appropriately to SH allegations are crucial to beliefs about reporting SHV; however, it may also be of importance for SH prevention. Perpetrators of SH are more likely to engage in harassing behaviors when contextual factors are favorable (Pina et al., 2009). For example, when men who score high on lenient attitudes toward SH (LSH) are provided with legitimate reasons to touch a woman, they engage in more attempts of sexual touching relative to low LSH men (Pina et al., 2009; Pryor, 1987). Men higher in LSH also engage in more SH behavior online—an environment with low accountability (Siebler et al., 2008). It was beyond the remit of this study to explore perpetrators’ motives for committing SH in the university context; however, universities should acknowledge the role that the environment has in facilitating SHV. Institutional intervention efforts should focus on increasing students’ awareness of what constitutes SH, reporting mechanisms for SHV, as well as the repercussions for engaging in SH behaviors. Future studies may then explore the incidence of SH in colleges that have adopted a visible zero tolerance stance to SH in comparison with those whose policies are unknown.

### Limitations

This study has several limitations that warrant discussion. We endeavored to explore the SHV experiences of minority groups; however, we were unable to conduct meaningful analyses among smaller intersecting groups. We also sought to explore the experiences of all students in Irish colleges, rather than the experiences of specific groups who have been documented in previous literature to be at greater risk of SHV (Wolff et al., 2017). We found that those who reported a physical disability were at an increased risk of SHV; however, we do not know if their disability was categorized as a visible or invisible disability. Those with visible disabilities may differ to those with invisible physical disabilities. In addition, because of the low numbers of individuals in certain disability groups, we combined certain disability groups to retain sufficient power. However, we do not know if this had an obscuring effect on the results. In terms of institutional support, although at risk groups were more likely to report lower scores of institutional support, it was beyond the remit of the study to explore if perceived university support related to lower wellbeing.

The current study employed the use of quantitative methodology. Quantitative studies cannot address the nuances involved in the intersection of SH. The use of interaction effects speaks to the quantity of an experience, not the quality of an experience (Bloom et al., 2021). This was an exploratory study of SHV in an Irish context, and it was beyond the remit of the study to explore the qualitative dimensions of our participants’ experiences of harassment. The type and severity of harassment will differ from one individual to another. This requires further research.

The current study used preexisting measures to assess SHV based on sexuality and gender. These measures have previously been validated among sample of predominantly white, heterosexual, and cisgender women (de Heer & Jones, 2017; Swartout et al., 2019). If researchers developed scales to explore sexual harassment experiences of alternate groups, they may produce different themes, factor structures, subscale categories, and items of relevance. For example, Collins (1990) refers to oppression, exploitation, and susceptibility to assault as core elements of black women's standpoint. Incorporating a black feminist or queer standpoint theory for the development of SHV measures, for example, may highlight different experiences that the current measure overlooks (Collins, 1990). Unfortunately, because the percentage of individuals who occupy some of these groups in society is in the minority, it is difficult to develop measures of statistical accuracy with some minority groups.

SHV was assessed by asking individuals to report their experience of sexual and gender-based harassment. However, a range of prejudices could have prompted a harasser to target any of the individuals in our sample. For example, previous studies show racialized sexual harassment may occur because of the stereotypical beliefs a person has about certain races or features that are believed to be racially distinctive (Buchanan & Ormerod, 2002; Cortina, 2001).

## Conclusion

In conclusion, the results from the current study suggest that some intersecting identities increase the likelihood for SHV during one's time in college in Ireland as elsewhere. Our findings support other research that has identified the association between minority category membership and SHV and that those most at risk of SHV perceive their college institutions as less supportive to victims of sexual harassment than those who have not experienced harassment. Our findings indicate that targeted intervention to support minority groups in HE in Ireland are needed to reduce the inequalities presented by SH victimization. Although this study presents a large sample in the Irish context, the majority of Irish students is white, heterosexual, young, able-bodied adults. Cross-cultural studies could facilitate more detailed analyses among minority ethnic, sexual orientation, and disability groups.
